# Supplementary material for: A noncanonical Pol III-dependent, Microprocessor-independent biogenesis pathway generates a germline enriched miRNA family
Source: bioRxiv. 2025 Jun 2:2025.04.11.648421. Preprint. [Version 2] doi: 10.1101/2025.04.11.648421 (PMC12157501; doi:10.1101/2025.04.11.648421)
Supplement: Supplement 1 [file media-1.pdf]

List of Supplemental Figures and Tables

Figure S1. Data related to Microprocessor-independence of *mir-1829.*

Figure S2. The germline-enriched *mir-1829* family is not required for fertility or viability.

Figure S3. Knock-in of *mir-1829b* transcriptional unit at heterologous locus generates mature *mir-1829b.*

Figure S4. The AID system allows for efficient depletion of DCR-1, RPC-1, and NSUN-2.

Figure S5. The *mir-1829* family is significantly reduced upon germline-specific Dicer depletion.

Figure S6. DRH-1, RDE-4, NSUN-2, and key cellular nucleases are dispensable for *mir-1829b/c* biogenesis.

Figure S7. Noncanonical miRNA biogenesis extends beyond the *mir-1829* family.

Table S1. Strains used in this study.

Table S2. Alleles generated and oligonucleotides used in this study.

Table S3. 5' RACE fragment positions relative to precursor.

Table S4. Samples used for deep sequencing.

Table S5. Raw miRNA and spike-in reads for all samples.

Table S6. Results of small RNA sequencing of Dicer knockdown in whole worms (soma & germline).

Table S7. Results of small RNA sequencing of Dicer knockdown in the germline.

Table S8. Expanded results of small RNA sequencing of annotated miRBase miRNAs in Dicer knockdown (soma & germline).

Table S9. Results of small RNA sequencing of RPC-1 knockdown in whole worms (soma & germline).

Table S10. Identification of additional noncanonical microRNAs.

Table S11. Summary of mis-annotated and noncanonical microRNAs.

Table S12. Expanded results of small RNA sequencing analysis of annotated miRBase miRNAs in germline-specific Dicer knockdown.

Table S13. Results of small RNA sequencing upon Pol III inhibition in iCas9-RKO cells.

Table S14. Results of small RNA sequencing after DGCR8 knockout in iCas9-RKO cells.

Table S15. Results of small RNA sequencing after DICER knockout in iCas9-RKO cells.

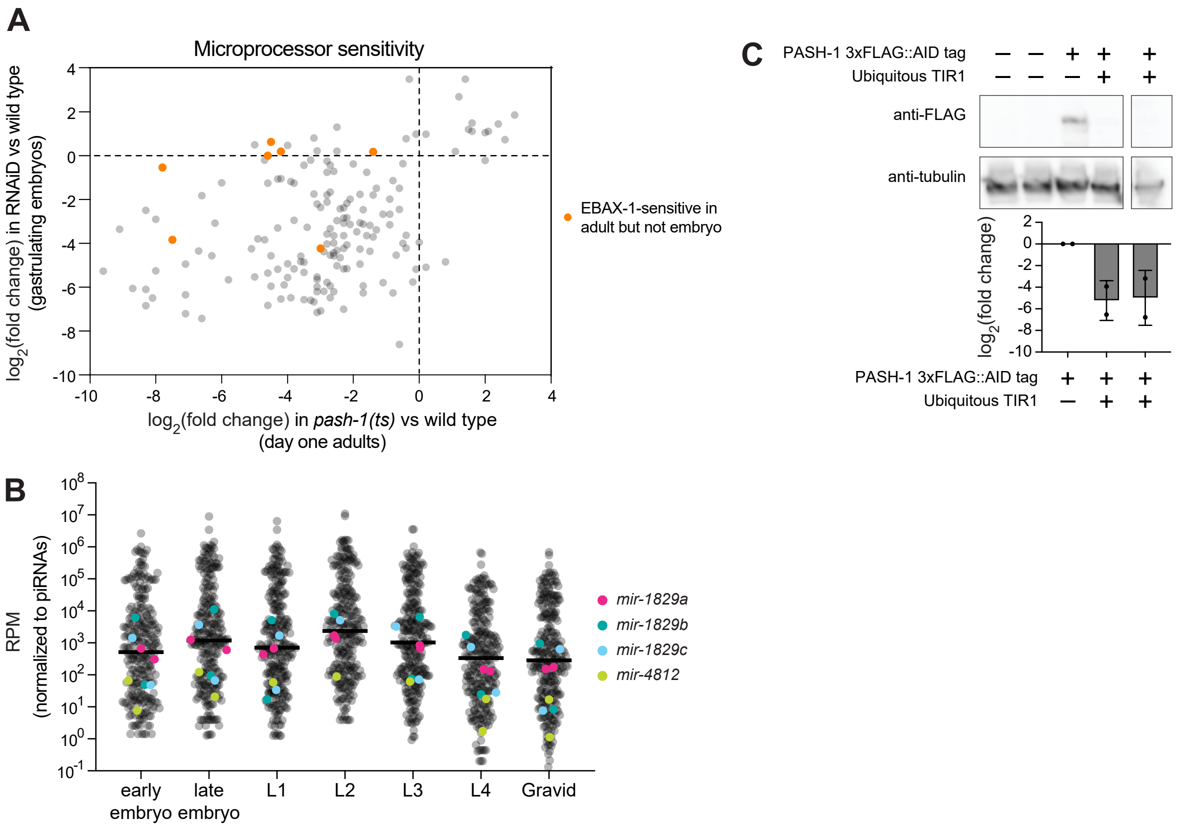

Figure S1. Data related to Microprocessor-independence of *mir-1829*. A) Comparison of $\log_2$(fold change) of miRNAs 24h post-upshift to restrictive temperature in *pash-1(ts)* vs. wild type (Vieux et al. 2021) (x-axis) to those in RNAiD depletion of the MP (Dexheimer et al. 2020) (y-axis) with highlighting of miRNAs that are stabilized in *ebax-1*$^{-/-}$ mutants in adults but not embryos. B) Relative abundance of *mir-1829* in relation to all miRNAs throughout *C. elegans* development from bias-minimized small RNA cloning from Stubna, et al. 2024. C) Representative western blot of PASH-1 knockdown using the AID system. Quantification of FLAG tag signal, normalized to tubulin signal, shown below. PASH-1 levels are normalized to PASH-1 AID tag without TIR1 (MLC1061) treated with auxin. Mean and SD shown.

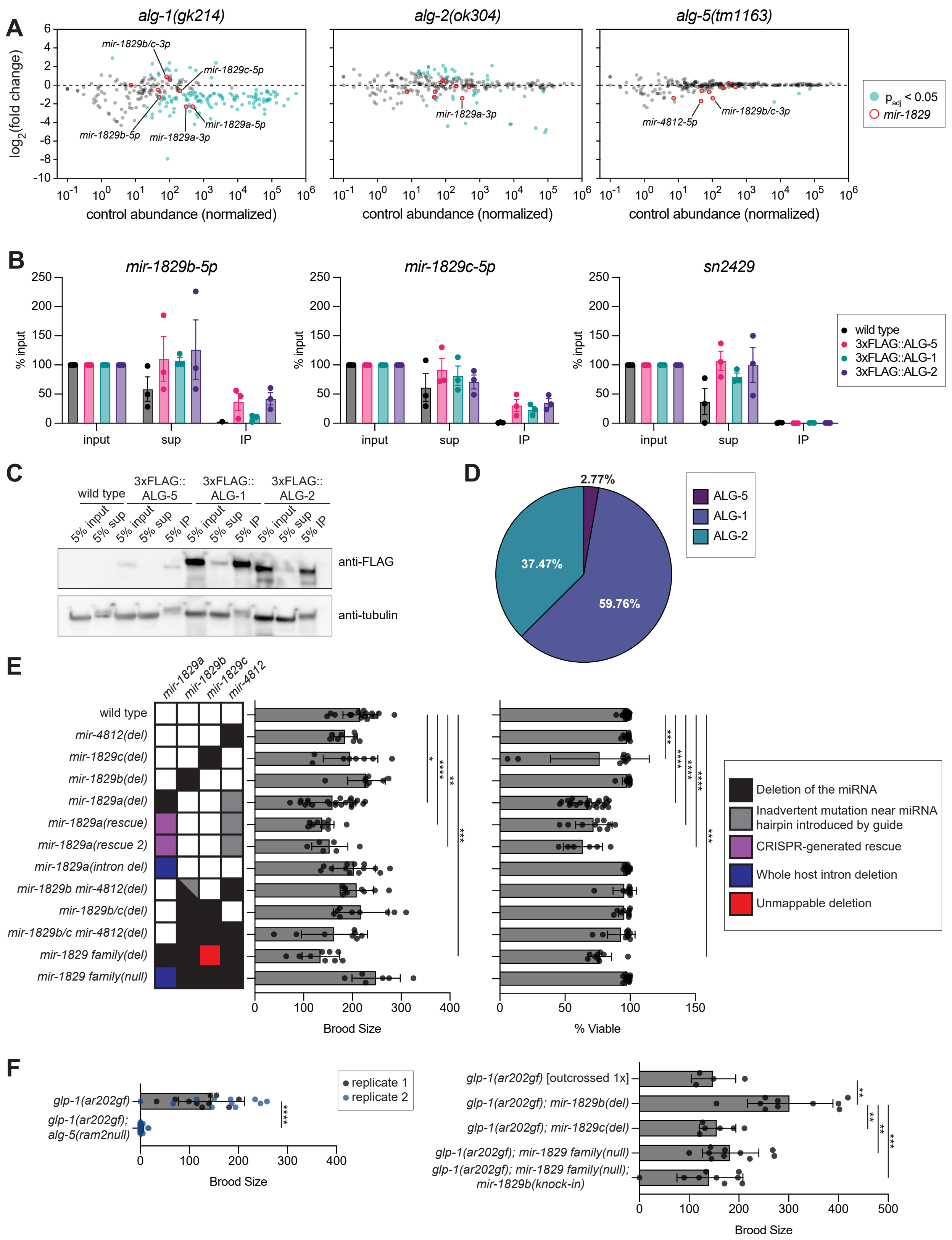

Figure S2. The germline-enriched *mir-1829* family is not required for fertility or viability. A) MA plots showing abundance in wild type on x-axis and $\log_2$(fold change) in Ago knockouts (*alg-1(gk214), alg-2(ok304), and alg-5(tm1163),* respectively) compared to wild type (N2) on y-axis

from Seroussi, et al. 2023. miRNAs showing significant sensitivity to Ago mutants are shown in blue-green, and the *mir-1829* family is highlighted in red open circles. Three biological replicates of each genotype were analyzed using DESeq2. B) RT-qPCR of *mir-1829b/c* levels in input, supernatant (sup), and immunoprecipitations (IP) of miRNA Argonautes ALG-1, ALG-2, and ALG-5. Wild type lysate and non-miRNA sn2429 were included as controls. *mir-1829b-5p* is evenly distributed between ALG-2 and ALG-5, and *mir-1829c-5p* is equally loaded into all three miRNA Argonautes. Mean and SEM shown. C) Representative western blot of miRNA Argonaute IPs used for RT-qPCR. 5% of input, supernatant (sup) and IP was loaded into each well. D) Representation of relative miRNA Argonaute abundances in whole animal adult samples. E) CRISPR-generated mutants of the *mir-1829* family were assessed for fertility (left) and embryonic viability (right) defects at 25°C. F) Consistent with Brenner *et al.*, *glp-1(ar202); alg-5(ram2)* double mutant hermaphrodites produce no progeny when upshifted from 15°C to 20°C (left). Mutations of the *mir-1829* family members do not phenocopy *alg-5(ram2)* in *glp-1(ar202)* background (right). (E-F) Mean and SD shown. Two-way ANOVA. Two-way ANOVA. ****p<0.0001, ***p<0.001, **p<0.01 *p<0.05.

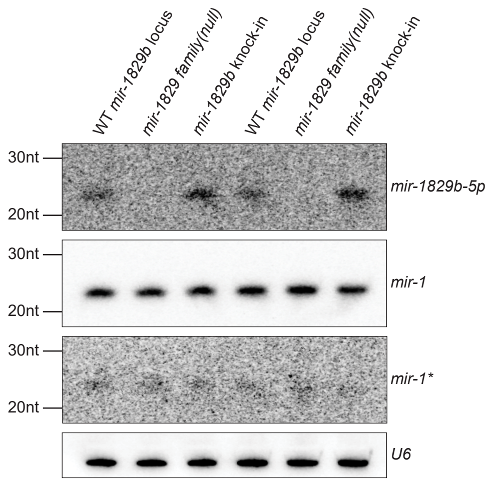

Figure S3. Knock-in of *mir-1829b* transcriptional unit at heterologous locus generates mature *mir-1829b.* Northern blot using low stringency conditions (see methods) demonstrates that re-integration of a 510bp minimal *mir-1829b* transcriptional unit in a *mir-1829 family(null)* mutant background expresses a mature small RNA (two biological replicates shown)

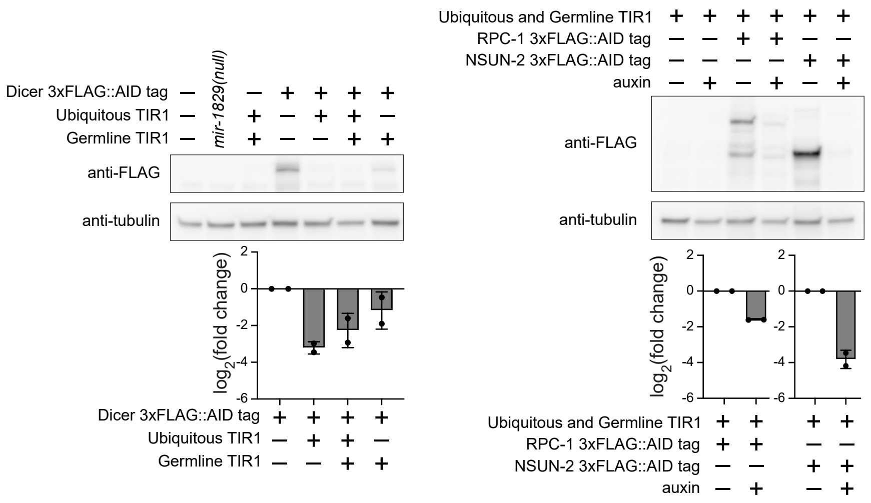

Figure S4. The AID system allows for efficient depletion of DCR-1, RPC-1, and NSUN-2. Representative westerns of Dicer/DCR-1 (left), RPC-1 and NSUN-2 knockdown (right) using the AID system. Quantification of FLAG tag signal normalized to tubulin signal shown below. Dicer levels are normalized to Dicer AID tag without TIR1 (UY212), and RPC-1 and NSUN-2 levels are normalized to samples not treated with auxin. Mean and SD shown.

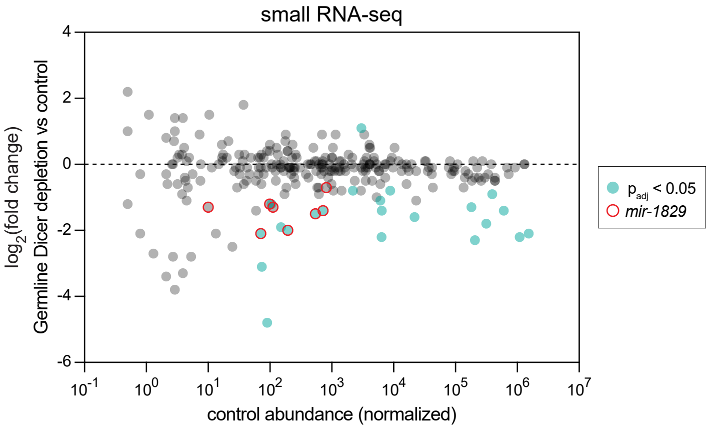

Figure S5. The *mir-1829* family is significantly reduced upon germline-specific Dicer depletion. Small RNA-seq. MA plot showing abundance in control (DCR-1::AID tag alone, UY212) on x-axis and $\log_2$(fold change) in DCR-1::AID; germline TIR1 (MCJ660) compared to UY212 on y-axis. miRNAs showing significant sensitivity to Dicer depletion are shown in blue-green, and the *mir-1829* family is highlighted in red open circles. Three biological replicates of each genotype were analyzed using DESeq2.

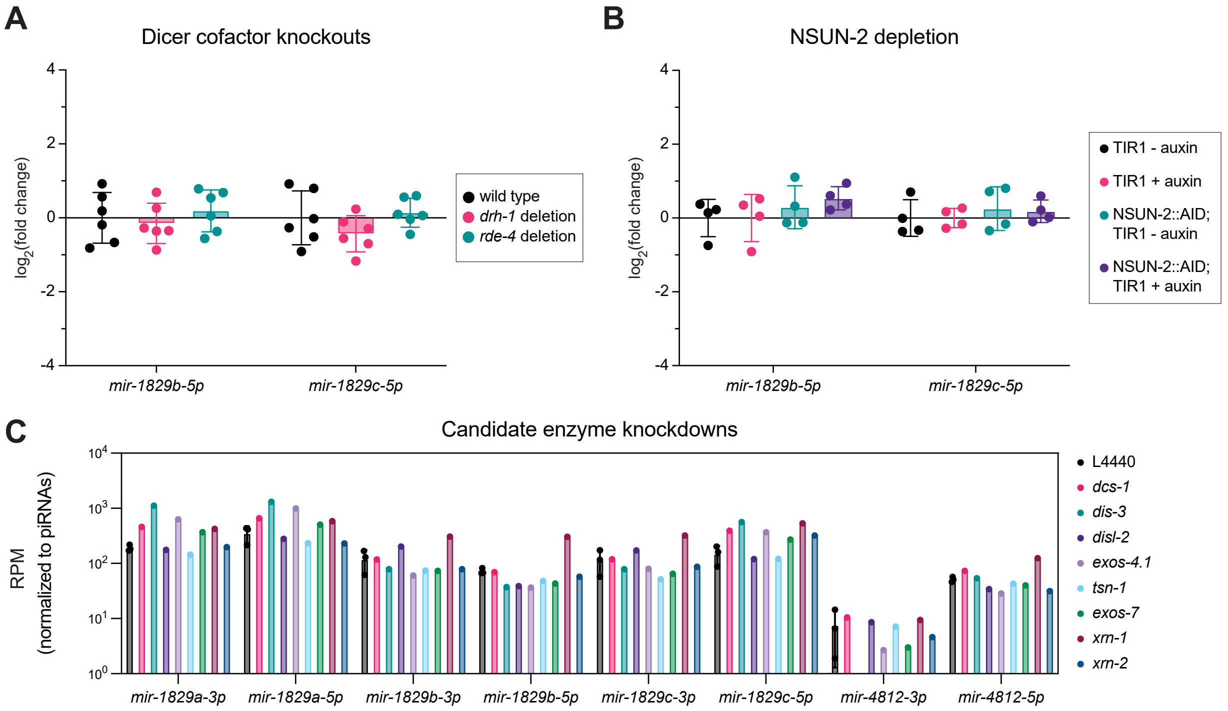

Figure S6. DRH-1, RDE-4, NSUN-2, and key cellular nucleases are dispensable for *mir-1829b/c* biogenesis. A) miRNA qPCR normalized to a small RNA control (*sn2429)* and further normalized to wild type levels. B) qPCR of miRNAs in AID-tagged NSUN-2 strain with ubiquitous TIR1 (MCJ677) or TIR1 alone (MLC1040). miRNA qPCR normalized to a small RNA control (*sn2429)*. MCJ677 without auxin treatment was further normalized to MLC1040 without auxin treatment, and auxin-treated MCJ677 was normalized to auxin-treated MLC1040. (A-B) Mean and SD shown. Two-way ANOVA showed no significant changes. C) Reads per million piRNA-mapping reads in adult samples grown on empty vector (L4440) or the indicated RNAi vector. Data was reanalyzed from Vieux et al. 2021.

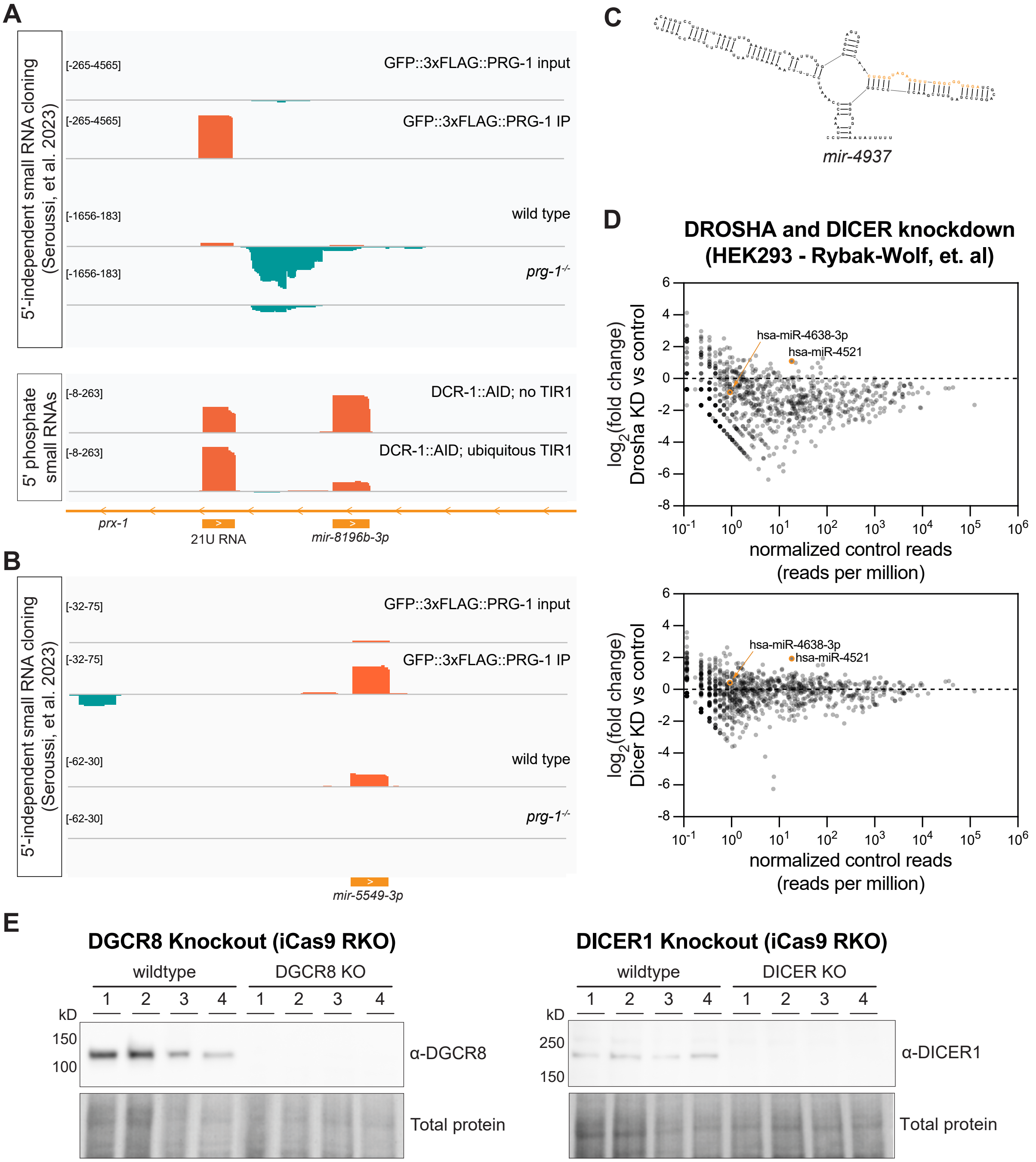

Figure S7. Noncanonical miRNA biogenesis extends beyond the *mir-1829* family. A) Genome browser tracks showing proximity of *mir-8196b* to an upstream recently-annotated 21U RNA. 5′-independent small RNA cloning following polyphosphatase treatment also shows 22G RNAs generated from the locus. *mir-8196b* but not the 21U shows Dicer-dependence. B) Genome browser tracks showing misannotated 21U RNA *mir-5549-3p,* which is enriched in PRG-1 IP and depleted in *prg-1*$^{-/-}$ mutants. C) Predicted secondary structure of *pri-mir-4937.* D) MA plot of small RNA sequencing from HEK293 cells in which DROSHA or DICER is knocked down by siRNA. *mir-4521* shows DROSHA- and DICER-independence (Rybak-Wolf, et al. 2014). E) Western blot demonstrating efficient knockout of DGCR8 or DICER1 in iCas9 RKO cells.
